# Safety and Efficacy of Amiodarone in a Patient With COVID-19

**DOI:** 10.1016/j.jaccas.2020.04.053

**Published:** 2020-05-29

**Authors:** Nadia Castaldo, Alberto Aimo, Vincenzo Castiglione, Cristiano Padalino, Michele Emdin, Carlo Tascini

**Affiliations:** a Azienda Sanitaria Integrata del Friuli Centrale, Udine, Italy; b Institute of Life Science, Scuola Sant’Anna Pisa, Pisa, Italy; c Cardiology Division, University Hospital of Pisa, Pisa, Italy; d Cardiology Division, Fondazione Toscana Gabriele Monasterio, Pisa, Italy

**Keywords:** amiodarone, COVID-19, SARS-CoV-2, CAD, cationic amphiphilic drug, COVID-19, coronavirus disease-2019, SARS-CoV-2, severe acute respiratory syndrome-coronavirus-2

## Abstract

There is an urgent need for effective treatments for coronavirus disease-2019 (COVID-19). Amiodarone, like hydroxychloroquine, exerts antiviral actions by interfering with endocytosis and viral replication. Here, to our knowledge, we report the first case of a patient affected by respiratory failure related to COVID-19 who recovered after only supportive measures and a short amiodarone course. (**Level of Difficulty: Beginner.**)

## Graphical abstract

**Figure:**
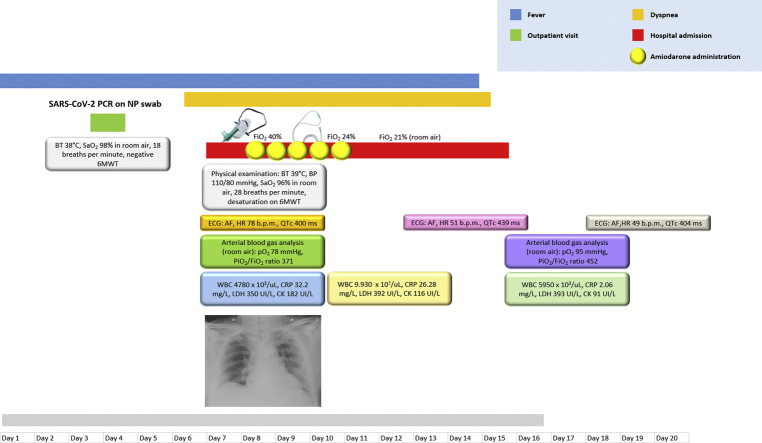

On March 23, 2020, a 74-year-old man presented to the emergency department of the University Hospital of Udine, Italy, because of fever, mild shortness of breath, and asthenia for 4 days.

**Learning Objectives**

• Amiodarone exerts antiviral actions by interfering with viral endocytosis and replication.
• In vitro, amiodarone hindered SARS-CoV-1 infection without modifying the density of angiotensin-converting enzyme 2 receptors on the cell surface.
• Amiodarone is a widely available, low-cost, relatively safe drug that could prove effective in modifying the early disease course of COVID-19.

## Past Medical History

The patient had a history of diabetes, hypertension, and permanent atrial fibrillation and was receiving therapy with metformin, perindopril, amlodipine, indapamide, bisoprolol, and dabigatran.

## Differential Diagnosis

Based on age, comorbidities, clinical presentation, and the current coronavirus disease-2019 (COVID-19) pandemic, the most plausible diagnosis seemed to be a severe acute respiratory syndrome-coronavirus-2 (SARS-CoV-2) infection.

## Investigations

A nasopharyngeal swab was collected on admission, and results were positive for SARS-CoV-2. The patient was febrile (38°C [100°F]), hemodynamically stable (arterial blood pressure: 110/80 mm Hg), with a good arterial oxygen (O_2_) saturation on room air (98%), an 18-breath/min respiratory rate, and no significant desaturation at the 6-min walking test. COVID-19 stage 1 was diagnosed according to staging system by Siddiqi et al. (1). The patient was prescribed symptomatic treatment with paracetamol and was discharged with recommendations for home self-monitoring.

During the following 4 days, the patient remained febrile (body temperature up to 39°C [102°F]) and became increasingly symptomatic for dyspnea. He was then admitted to the Infectious Disease Unit, where he was found pale, tachycardic (mean heart rate 87 beats/min, with a corrected QT interval [QTc] of 400 ms), with a high respiratory rate (28 breaths/min), a 96% O_2_ saturation on room air decreasing to 92% during the 6-minute walking test. His arterial blood gas analysis on room air showed moderate hypoxemia (Po_2_, 78 mm Hg) and a ratio of arterial oxygen *partial pressure* to fractional inspired oxygen (Pio_2_/Fio_2_ ratio) of 371 (normal value: >500). Lung ultrasonographic examination showed apical pleural line irregularities and bilateral areas of white lung. Accordingly, chest radiography revealed bilateral interstitial pneumonia.

## Management

The patient was placed on O_2_ therapy through a face mask with 40% Fio_2_. Intravenous steroid treatment with 8 mg dexamethasone twice a day was started but was stopped after 2 days because of significant increase in blood glucose despite insulin therapy. Given that the patient had no contraindications to amiodarone and gave his informed consent, off-label therapy with amiodarone was started on the second day from admission. The drug was administered on day 1 as a 15-mg/kg/24 h intravenous infusion, followed by the oral administration of 400 mg twice daily. This dosage was chosen to achieve a serum concentration higher than 10 μmol/l without exceeding 20 mg/kg/day, which is the maximal suggested dose in patients with heart diseases (2). Plasma amiodarone concentration, measured on day 3, was 0.55 μg/ml (reference interval: 0.21 to 2.05 μg/ml).

**Figure 1:**
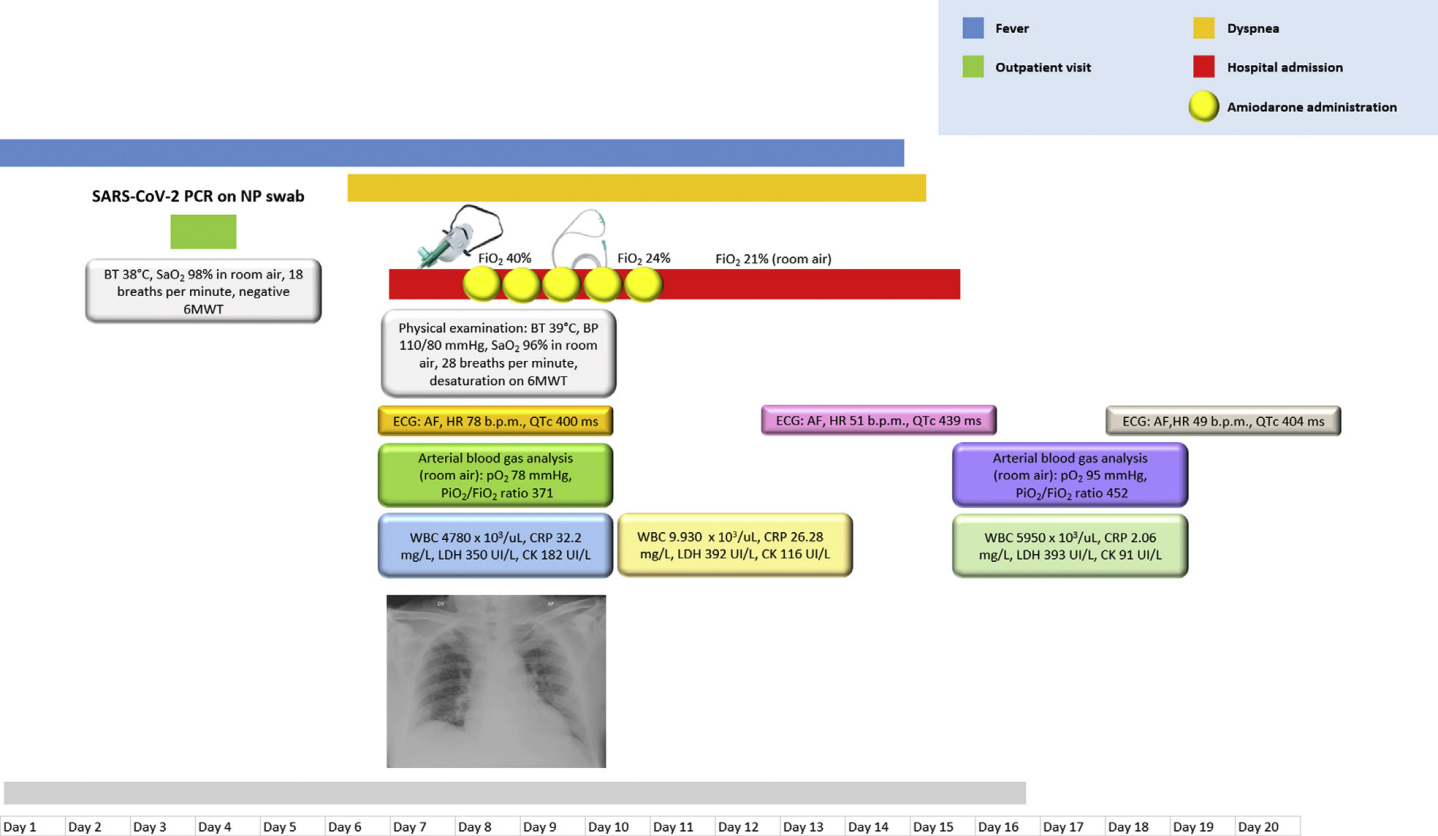
Clinical Course Findings from physical examination, electrocardiogram (ECG), arterial blood gas analysis, and laboratory examinations. 6MWT = 6-min walking test; AF = atrial fibrillation; BP = blood pressure; b.p.m = beats per minute; BT = body temperature; CK = creatine kinase; CRP = C-reactive protein; Fio_2_ = fractional inspired oxygen; HR = heart rate; LDH = lactate dehydrogenase; NP = nasopharyngeal; Pio_2_ = arterial oxygen partial pressure; PCR = polymerase chain reaction; Sao_2_ = oxygen saturation in the arterial blood; WBC = white blood cells.

Tables 1 and 2 summarize the biochemical tests and atrial blood gas analysis before, during, and after amiodarone therapy. Figure 1 shows the illness clinical course.

**Table 1:** Biochemical Test Results Before, During, and After Amiodarone Therapy

	Before Amiodarone	During Amiodarone (Day 2)	After Amiodarone	Reference Values
WBC, ×10^3^/μl	4.780	9.930	5.950	4.00–11.00
Lymphocytes, ×10^3^/μl	0.490	0.880	1.620	1.00–4.00
Platelets, ×10^3^/μl	171	247	214	150–400
CRP, mg/l	32.2	26.28	2.06	0.00–5.00
PCT, ng/ml	0.04	0.05	0.06	<0.10
INR	1.27	—	1.11	0.85–1.15
D-dimer, ng/ml	150	—	206	0–500
LDH, IU/l	350	392	393	240–480
CK, IU/l	182	116	91	39–190

CK = creatine kinase; CRP = C-reactive protein; INR = international normalized ratio; LDH = lactate dehydrogenase; PCT = procalcitonin; WBC = white blood cells.

**Table 2:** Arterial Blood Gas Analysis Results Before and After Amiodarone Administration

	Before Amiodarone	After Amiodarone
pH	7.48	7.46
Pco_2_, mm Hg	38	36
Po_2_, mm Hg	78	95
HCO_3_^–^, mEq/l	28.6	25.6
Fio_2_, %	21	21
Po_2_/Fio_2_ ratio	371	452

FiO_2_ = fractional inspired oxygen; HCO_3_^–^ = bicarbonate level; pCO_2_ = arterial carbon dioxide arterial oxygen *partial pressure*; pO_2_ = arterial oxygen *partial pressure*.

Therapy with amiodarone lasted 5 days, during which the patient’s symptoms improved and fever disappeared. Biochemical analysis also showed a decrease of C-reactive protein and lactate dehydrogenase and an increase in leukocytes. Serial arterial blood gas analyses revealed a progressive improvement of respiratory function, and O_2_ supplementation became unnecessary on day 4 after admission. The electrocardiogram was checked daily. The QTc interval was prolonged up to 439 ms on day 8 from admission. However, 6 days after drug withdrawal, the QTc interval returned to baseline level (404 ms). No adverse events were reported. The patient was discharged on day 8.

## Discussion

As of April 27, 2020, almost 3 million cases of infection and almost 207,000 deaths by the novel coronavirus SARS-CoV-2 have been reported. The lack of a standardized treatment strategy for COVID-19 has prompted assessment of the possibility of repurposing older drugs, such as hydroxychloroquine, for new indications (3).

Hydroxychloroquine belongs to the class of cationic amphiphilic drugs (CADs) that are characterized by a hydrophobic aromatic ring or ring system and a hydrophilic side chain containing an ionizable amine functional group (4). Because of their structure, CADs accumulate in acidic compartments, such as late endosomes/lysosomes, reducing their luminal acidity, altering the trafficking of membrane components, and inducing in several cell types, such as example alveolar macrophages, a Niemann-Pick disease type C–like phenotype (4). This may affect cell activities important for an efficient viral internalization, such as partial hydrolysis of viral surface proteins, macro- and/or micropinocytosis, the organization of the membrane invagination systems, and vesicular transport of material to the lysosomes (4).

The category of CADs includes antidepressants, antibiotics, antipsychotics, cholesterol-lowering and fertility-regulator drugs, and antimalarial medications. Several CADs with antiarrhythmic properties have proven effective against RNA viruses in vitro; most notably, dronedarone, verapamil, and the calcium channel blocker bepridil inhibited filovirus infection in cell cultures and mouse models (4,5). Amiodarone is a widely available, low-cost antiarrhythmic drug that in the past has been considered as a possible antiviral medication (6). Amiodarone and its main metabolite (mono-*N*-desethyl amiodarone) were shown to inhibit the entry of filoviruses (a family of single-stranded, negative-sense RNA viruses that includes Ebola virus) at the same serum concentration found in patients treated for arrhythmias (7,8). Amiodarone also proved able to block the spreading of SARS-CoV-1 infection in cell cultures without modifying the density of angiotensin-converting enzyme 2 receptors on the cell surface or interfering with the attachment of SARS-CoV-1 to the cells (9). Interestingly, amiodarone displayed antiviral activity even when SARS-CoV-1 delivered its genome directly into the cytoplasm, bypassing the endocytic compartment (9). Therefore, although the antiviral activity of amiodarone is most likely due to interference with the endocytic pathway, further mechanisms cannot be excluded.

Based on this evidence, it can be postulated that amiodarone administration in an early disease phase might block SARS-CoV-2 replication. The safety and efficacy of amiodarone in patients with COVID-19 remain to be investigated, and drug interaction with other treatments (e.g., hydroxychloroquine, lopinavir/ritonavir, atazanavir, and darunavir/cobicistat) are major concerns (10). Notably, amiodarone toxicity is mainly cumulative and dose dependent, which means that the risk of adverse events is directly related to the duration of the treatment (11). Therefore, a short amiodarone course could be proposed as a stand-alone medication in cases of mild to moderate COVID-19 and could be hypothetically administered with interacting drugs under strict drug monitoring.

Here, to our knowledge, we report the first case of a patient affected by respiratory failure related to COVID-19 who recovered after only supportive measures and a short amiodarone course. We must acknowledge that we do not know whether the clinical evolution would have been the same if the patient had received other drugs used in patients with COVID-19 and to what extent amiodarone effectively changed the natural course of the disease. Despite these limitations, this case points to amiodarone as a possible therapy for the early phase of COVID-19. A randomized trial comparing amiodarone and another CAD (verapamil) with usual care in hospitalized patients with confirmed COVID-19 has been started (Amiodarone or Verapamil in COVID-19 Hospitalized Patients With Symptoms [ReCOVery-SIRIO]; NCT04351763). In this study, amiodarone will be administered as a bolus of 150 mg over ≥10 min, followed by continuous infusion of 1 mg/min for 6 h, then 0.5 mg/min for 18 h, and finally oral administration of 200 to 400 mg daily (based on cardiac response and age) until discharge. The primary endpoint will be clinical improvement (assessed on a scale ranging from 1 to 7) from randomization to day 15. Secondary endpoints will include time to resolution of fever, tachyarrhythmias, duration of hospital stay, and mortality.

## Follow-Up

At follow-up visit, 10 days after discharge, the patient was asymptomatic.

## Disclaimer

In this case, amiodarone was given for research purposes in a hospital setting. This is an approach still under investigation. Do not try this at home.

## Conclusions

The current COVID-19 pandemic has prompted the urgent need for effective treatments. Repurposing old drugs with new indications could be an effective option. Amiodarone, like hydroxychloroquine, exerts antiviral actions by interfering with viral endocytosis and replication. Given its wide availability, low cost, and relatively safe profile, it should warrant consideration as a possible therapy for the early phase of COVID-19.
